# Simple extraction methods for pesticide compound-specific isotope analysis from environmental samples

**DOI:** 10.1016/j.mex.2022.101880

**Published:** 2022-10-13

**Authors:** Tetyana Gilevska, Charline Wiegert, Boris Droz, Tobias Junginger, Maria Prieto-Espinoza, Adrien Borreca, Gwenaël Imfeld

**Affiliations:** Université de Strasbourg, CNRS/ENGEES, ITES UMR 7063, Institut Terre et Environnement de Strasbourg, 5 Rue René Descartes, 67000 Strasbourg, France

**Keywords:** Pesticide extraction, stable isotopes, CSIA, degradation, transformation products

## Abstract

Compound-specific isotope analysis (CSIA) is a powerful approach to evaluate the transformation of organic pollutants in the environment. However, the application of CSIA to micropollutants, such as pesticides, remains limited because appropriate extraction methods are currently lacking. Such methods should address a wide range of pesticides and environmental matrices, while recovering sufficient mass for reliable CSIA without inducing stable isotope fractionation. Here, we present simple extraction methods for carbon and nitrogen CSIA for different environmental matrices and six commonly used herbicides, i.e., atrazine, terbutryn, acetochlor, alachlor, butachlor, and *S-*metolachlor, and three fungicides, i.e., dimethomorph, tebuconazole, and metalaxyl. We examined the potential of several extraction methods for four types of soils or sediments, three types of environmental waters and aerial and root plant samples for multielement (ME)-CSIA.

• Pesticide extraction recoveries varied depending on the physical characteristics of the pesticides and matrix properties for environmental water (77 to 87%), soil and sediment (35 to 82%), and plant (40 to 59%) extraction.
• The tested extraction methods did not significantly affect the carbon and nitrogen stable isotope signatures of pesticides (Δ(^13^C) <0.9‰ for Δ(^15^N) <1.0‰).

## Highlights

• Extractions from soil, plants, and water were tested for pesticide C and N CSIA.
• Pesticide recoveries strongly varied among compounds and matrices properties.
• Tested extraction methods caused no effect on δ^13^C and δ^15^N of pesticides.
• C and N pesticide CSIA can be applied *in situ* to agricultural water samples.
• Pesticide CSIA for soil and sediment samples are limited to source areas.

## Graphical abstract

**Figure:**
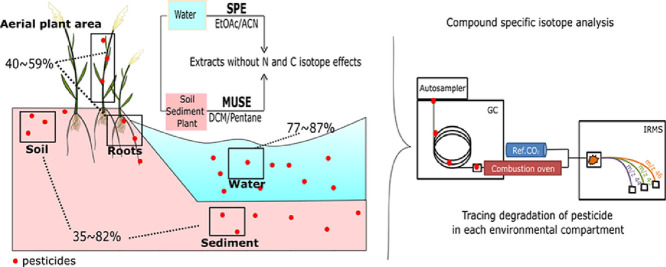

Specifications Table

**Table:** 

Subject Area:	Environmental Science
More specific subject area:	Compound-specific isotope analysis
Method name:	Extraction methods for pesticide compound-specific isotope analysis from environmental samples
Name and reference of original method:	Modified ultrasonic-assisted extraction (MUSE) Quick, Easy, Cheap, Effective, Rugged and Safe procedure (QuEChERS) Solid- phase extraction (SPE) [1], [2], [3]
Resource availability:	Gas chromatography/isotope ratio mass spectrometer (GC/IRMS)

## Background

Compound-specific isotope analysis (CSIA) helps to identify degradation pathways of pesticides and even to estimate the extent of pesticide degradation [4]. A multielement (ME)-CSIA approach enables the elucidation of reaction pathways in complex pollution scenarios, such as multiple contamination sources or concurrent degradation processes. However, the applications of ME-CSIA for pesticides require validated and efficient extraction methods from environmental matrices. Extraction methods for pesticide residues from environment matrices for ME-CSIA should: (i) provide sufficient analyte mass for reliable isotope analysis, (ii) cause no stable isotope effect (Δ(^H^E)), (iii) be applicable to a wide range of pesticides and matrices, and (iv) limit matrix co-enrichment to avoid co-elution during chromatographic separation.

Previously, solid-phase extraction (SPE) for pesticide extraction from water for CSIA has been tested for atrazine, acetochlor, *S-*metolachlor, metalaxyl, butachlor, alachlor, terbutryn, chlordizon, and several of their transformation products [5], [6], [7], [8]. Pesticide extraction methods from soil, sediment and plants for reliable pesticide CSIA have been used in both laboratory and field studies [9], [10], [11], [12]. Ivdra et al. [2] proposed a modified ultrasonic-assisted extraction (MUSE) method without carbon isotope effect (Δ(^13^C) ≤0.4‰). Other studies used accelerated solvent extraction (ASE) for the extraction of hexachlorocyclohexanes (HCHs) from soil and plants for C, H, and Cl isotopes analysis [12]. The application of Quick, Easy, Cheap, Effective, Rugged and Safe procedure (QuEChERS) for the extraction of metolachlor from two agricultural soils led to a systematic but reproducible isotope fractionation for Cl (Δ(^37^Cl) between +2.5 and +3.5‰) [13].

In this context, the purpose of this study was to test and compare simple extraction methods for pesticide ME-CSIA from various environmental matrices, with emphasis on carbon (*δ*^13^C) and nitrogen (*δ*^15^N) values. Efficient pesticide extraction from diverse matrices can improve the use of ME-CSIA to assess pesticide degradation and help develop monitoring and remediation strategies. To develop an overview for the application of pesticide CSIA extraction methods four types of soils and sediments, three types of environmental waters, and aerial parts and roots of plants were tested. We selected six commonly used herbicides, i.e., atrazine, terbutryn, acetochlor, alachlor, butachlor, and *S*-metolachlor, and three fungicides, i.e., dimethomorph, tebuconazole, metalaxyl, representing a wide range of physicochemical properties and belonging to four chemical families (Table S1). Here, we extend previous findings for SPE and ME-CSIA to new pesticides and environmental matrices. We further tested a MUSE method for extracting selected pesticides from soil, sediment, and plant material, thus paving the way for wider application of ME-CSIA of micropollutants in the environment.

## Chemicals

Extraction solvents: dichloromethane (DCM), acetonitrile (ACN), ethyl acetate (EtOAc), pentane, methanol (MeOH)) were HPLC grade purity (>99.9%) and purchased from Sigma–Aldrich (St. Louis, MO, USA). Analytical standards (purity >98%) atrazine, terbutryn, acetochlor, alachlor, butachlor, *S*-metolachlor, *S*-metolachlor d-11, dimethomorph, tebuconazole, and metalaxyl were PESTANAL grade purchased from Sigma–Aldrich (St. Louis, MO, USA).

## Sampling and characterization of water, soil, sediment, and plants

### Water

Three types of water matrices were tested: (i) buffered (pH=7) ultrapure water (>18 MΩ cm), (ii) storm water from a vineyard catchment (Rouffach, France) [14], (iii) runoff and river water from two different crop catchments (Alteckendorf and Souffel, France) [3,10]. Field samples were successively filtered at 11 µm trough grade one cellulose (Whatmann 1001-047) and 0.45 µm cellulose acetate membrane filters, and stored at 4°C until further analysis. The hydrochemical composition (Table S2) was analyzed by ionic chromatography (IC) and inductively coupled plasma atomic emission spectroscopy (ICP-AES) using standard analytical procedures (NF/ISO), as described elsewhere [15].

### Soil and sediment

The top 0−10 cm of i) river sediments from the Souffel river, France, [14], ii) vineyard soils from the Rouffach catchment [10], iii) vineyard storm water sediments (Rouffach, France [14]), iv) forest soils (Strengbach, France [10]) were sampled with a shovel cleaned with ultrapure water and ethanol and wiped between collections. Soil and sediment samples were sieved through a 2-mm mesh and stored at 4°C before spiking. The physicochemical characteristics of the sieved soil and sediment samples (Table 1, Table S3) were measured using standard analytical procedures (NF/ISO) [15].

**Table 1:** Physicochemical properties of tested soils and sediments (see also Table S3), mean value ± standard deviation (1 σ) for all measurements.

	Clay [%]	Silt [%]	Sand [%]	Organic carbon [%]	pH	Water content [%]	Cation exchange capacity (CEC) [cmol^+^/kg]
Forest soil, Strengbach	7 ± 0.3	32 ± 1	62 ± 2	4.5 ± 0.2	3.4 ± 0.1	47 ± 12	14
Vineyard soil, Rouffach	23 ± 2	68 ± 9	9 ± 7	1.1 ± 0.2	7.9 ± 0.1	13 ± 7	18
River sediment, Alteckendorf	14 ± 0.4	73 ± 2	13 ± 0.4	2.0 ± 0.2	7.8 ± 0.1	39 ± 3	15
Storm water sediment, Rouffach	24 ± 8	45 ± 19	32 ± 22	2.5 ± 0.4	7.6 ± 0.1	41 ± 6	14

### Plants

Mature common reed (*Phragmites australis* Cav. Trin. ex Steud., 1840) individuals (*n*=20) were sampled from the Rouffach storm water wetland, separated into roots and aerial parts, which were extracted separately. Plant material was washed by shaking the roots and rhizosphere for 10 min in ultrapure water. The water was replace several times until all the sediment rhizosphere was separated from the roots [16]. A 0.9 % NaCl solution was used to rinse twice the plant material to remove rhizosphere and rhizoplane sediment. Plant material was homogenized with a hand blender (Bosch MSM66110) and frozen at –20°C until further use.

## Pesticide extraction from water

Pesticide stock solutions (1 g/L) of pesticides in DCM or ACN were added to buffered (13.6 g/L monopotassium phosphate, 4 g/L sodium hydroxide) ultrapure water or environmental water and stirred until complete solvent evaporation to reach concentrations ranging from 0.5 µg/L to 6 mg/L. The extraction method was adapted from USA EPA method 525.2 using an AutoTrace 280 SPE system (Dionex®, CA, USA) for the simultaneous extraction of 6 samples [3] (details in SI). Tested water volumes were: 0.01, 0.5, 1, 2, 4, and 8 L.

## Soil, sediment and plant extraction methods

### MUSE method

Pesticides in sediment, soil or plant were extracted using a solid-liquid extraction protocol adapted from [1,2]. Five grams of soil or sediment (dry weight) were placed in an amber glass centrifuge tube. Due to the large (∼85%) water content of plants, 3 g of plant dry weight was used for extraction. Sediment samples were all extracted keeping 100% of water content. Triplicate or duplicate samples were spiked (0.5 to 50 µg/g) with aqueous solutions of pesticides, vortexed, shaken (orbital shaker 80 revolutions per minute (rpm)) for at least 30 min, and incubated in the dark for three days at 4°C. 1 mL of EtOAc or DCM:pentane (3:1) per gram of sample was then added and vortexed for 5 s, followed by 5 min in an ultrasonic bath (Branson 5510, 40 kHz) for homogenization. The sample was vortexed for 1 min, followed by centrifugation (2400 rpm) for 20 min. The supernatant was transferred to an amber glass vial, and the extraction method was repeated two more times. The supernatants were pooled and concentrated at room temperature under a gentle nitrogen stream to the last drop, before resuspension into ACN to a volume of 1 mL by vortexing (5 s) and ultrasonication (5 min) to collect pesticide residues. Then 75 mg of anhydrous magnesium sulfate (MgSO_4_) was added to remove residual water and 13 mg of primary-secondary amine (PSA bonded silica, Supelco P/N 52738) as a clean-up agent [1]. The vial was vortexed for 30 s, centrifuged at 2400 rpm for 5 min, and the supernatant was transferred to a clean amber glass vial for further analysis.

### Two-step extraction method for hydrophilic soil or sediment

We tested a two-step extraction from soil and sediment when the extraction yield with DCM:pentane (3:1) was low, due to limited solvent penetration in the soil or the sediment. Recovery with DCM:pentane (3:1) in such case was not improved by prolonged vortexing or shaking, possibly due to the hydrophilic nature of organic carbon in the sediment, containing high proportion of polar carbon species (e.g., C–OH, C=O [17]). In the first extraction step, 10 mL of 0.01 M calcium chloride (CaCl_2_) was added to 5 g of soil or sediment (dry weight). Samples were vortexed for 5 s, ultrasonicated for 5 min, vortexed again for 1 min, and centrifuged for 10 min at 2000 rpm before collecting 10 mL of the water fraction. The extraction was repeated twice to reach a final fraction of 30 mL of the aqueous soil extract. The remaining soil and sediment fraction was extracted in a second step using the above-described MUSE method with MeOH as an extraction solvent. While the 30 mL water supernatant fraction was extracted following a liquid-liquid extraction method by adding 3 mL of DCM, followed by vortexing for 5 min, ultrasonication for 5 min and centrifugation for 5 min (1500 rpm). Next, the solvent (DCM) was collected from the bottom of the vial. This step was repeated three times, yielding a final solvent fraction of 9 mL. The solvent extract was evaporated to dryness and resuspended in ACN, vortexed for 30 s, ultrasonicated for 5 min, and transferred into a GC vial for concentration analysis and CSIA.

## Pesticide quantification

Pesticides were analyzed with a gas chromatograph (GC, Trace 1300, ThermoFisher Scientific) coupled to a mass spectrometer (MS, ISQ™, ThermoFisher Scientific). Chromatographic separation was performed with a TG –5MS column (30 m × 0.25 mm ID, 0.25 µ film thickness). *S*-metolachlor d-11 or atrazine d-5 were used as internal standards and injected with every injection to account for reproducibility of the autosampler. Additional parameters are described in SI (Table S4). All samples were diluted to be within the linear calibration range (0.1 to 1 mg/L).

## Pesticide CSIA

Carbon ($\delta^{13}C)$ and nitrogen ($\delta^{15}N)$ stable isotope signatures of pesticides were measured using a GC-C-IRMS system consisting of a TRACE™ Ultra Gas Chromatograph (ThermoFisher Scientific) coupled via a GC IsoLink/Conflow IV interface to an isotope ratio mass spectrometer (DeltaVplus, ThermoFisher Scientific). Chromatographic separation was performed on a TG –5MS column (60 m × 0.25 mm ID, 0.25 µ film thickness). Samples were injected in split/splitless modes with an injection volume of 2 µL and injector temperature of 250°C. Samples were injected in triplicates and $\delta^{13}C$ values are reported as the arithmetic mean. For both elements, target compounds were combusted to CO_2_ or N_2_ in a single combined reactor (P/N 1255321, NiO tube and CuO-NiO-Pt wires, Thermo Fischer Scientific) at 1000°C. For N, liquid N_2_ was used for cryogenic trapping of CO_2_. Laboratory BTEX (Benzene, Toluene, Ethylbenzene and o-Xylene, carbon) and caffeine (nitrogen) standards were injected at the beginning of each session to check the performance of the instrument. Isotope values of pesticide standards were calibrated relative to Vienna Pee Dee Belemnite (VPDB) and AIR scales with EA–IRMS (Flash EA IsoLink^TM^ CN IRMS, Thermo Fisher Scientific) using a two‐point calibration with international reference materials AIEA600, USGS40, and USGS41. Standard injection with known isotopic signatures of in-house standards was injected at least every six samples for carbon and every three samples for nitrogen to follow up the measurement accuracy. The reproducibility of *δ*^13^C and *δ*^15^N measurements ranged from ± 0.4 to ± 0.6 ‰ and from ± 0.4 to ± 0.5 ‰, respectively. The total uncertainly, including accuracy and reproducibility, ranged, respectively from ± 0.6 to ± 0.8 ‰ and from ± 0.6 to ± 0.7 ‰, respectively. All isotopic measurements of element E (C and N) are reported in delta notation:

(1) $$\delta {}^{H}E=\left[\frac{{\left(\frac{{}^{H}E}{{}^{L}E}\right)}_{sample}}{{\left(\frac{{}^{H}E}{{}^{L}E}\right)}_{standard}}-1\right]\times \;1000$$

where *δ*^H^E is the isotopic value (‰),$\;{\left(\frac{{}^{H}E}{{}^{L}E}\right)}_{sample}$ is the ratio of heavy to light isotopes in the sample, and ${\left(\frac{{}^{H}E}{{}^{L}E}\right)}_{standard}$is the ratio of heavy to light isotopes in the standard in an international isotopic scale (Vienna Pee Dee Belemnite for carbon and AIR for N).

The method detection limit (MDL) was defined as the lowest concentration within a linear interval of ± 0.6‰ of the standard's mean value with good reproducibility (1σ<0.6‰, n≥ 3) .

## Method validation

### Extraction from water

#### SPE recoveries

**Figure 1:**
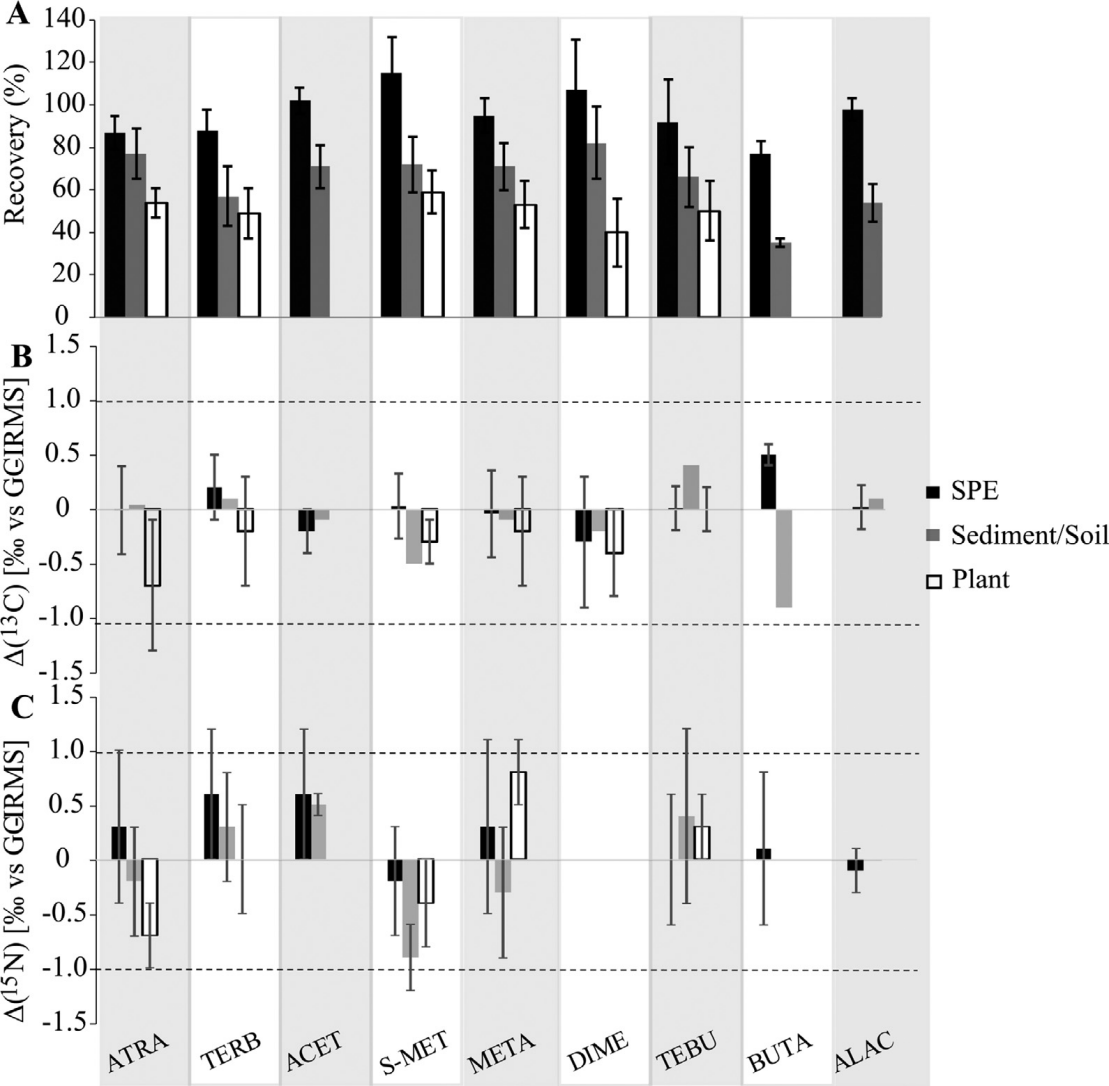
A - Extraction recovery, B – Effect on *δ*^13^C (Δ(^13^C) _[‰ vs GC-IRMS]_), C – Effect on *δ*^15^N (Δ(^15^N) _[‰ vs GC-IRMS]_) of SPE (water samples), and MUSE with DCM:Pentane (3:1) (sediment, soil, plant) for pesticides and all matrices combined. ATRA – atrazine, TERB – terbutryn, ACET – acetochlor, S-MET – S-metolachlor, META – metalaxyl, DIME – dimethomorph, TEBU – tebuconazole, BUTA – butachlor, ALAC – alachlor. Error bars denote standard deviation (1 σ, n ≥ 8). Dashed lines represent ± 1‰ (significance threshold) from reference isotope values of in-house standards measured with GC-IRMS.

The mean extraction recovery of pesticide for SPE ranged from 77 ± 8 % (butachlor) up to 115 ± 12 % (*S*-metolachlor) (Fig. 1, Table S5) across all conditions. Extraction recoveries for atrazine, acetochlor, *S*-metolachlor, metalaxyl, and alachlor were similar than those previously obtained for different sorbents and amounts [5,6,8,15]. Overall, octanol-water partition constant of pesticides (log *K_ow_*; Table S1) did not correlate with pesticide recoveries. Lower extraction efficiency (77%) of butachlor, in agreement with previous results [8], may be related to the longer alkoxy chain and the 2-carbon alkyl chain bound to the aromatic ring. Indeed, the log *K_ow_* of butachlor is higher than that of the other compounds, indicating an apolar character. Hence, ACN and EtOAc may not be the optimal choice for butachlor (Table S1), and more apolar solvents, such as DCM, may be more appropriate for the extraction of apolar pesticides (Fig. 4). Furthermore, physicochemical properties of parent compounds and their transformation products (TPs) may largely differ, implying that different sorbents or sorbent combinations are required to pre-concentrate both the parent compounds and the TPs [5].

Lower extraction recoveries for pesticides during SPE may be due to the breakthrough effect, i.e., the saturation of the sorbent capacity by the environmental matrix or washing away of sorbed compounds with water pumped through the cartridge [6]. No breakthrough effect was observed with increasing concentration or volume (Fig. S1 and Fig. S2) and for the different matrices (Fig. S3). This indicates that environmental matrix, i.e. nonvolatile matrix components co-enriched with the target compounds during the extraction did not cause mass loading or lower extraction recovery. While SPE was conducted using one gram of sorbent (SolEx C18, Dionex®), increasing the mass of the sorbent up to 10 g may increase the extraction recovery and allow the extraction of larger volumes and/or heavily contaminated samples in point-source areas [5].

#### Effect of SPE on stable isotope signatures

Overall, our results show that SPE can be effectively used for the pre-concentration of tested pesticides prior to carbon and nitrogen CSIA. Both carbon and nitrogen isotopes signatures of pesticides were not affected by the matrix, volume or concentration of tested pesticides (Δ(^13^C) <0.9‰ for Δ(^15^N) <1.0‰), p>0.05, Kruskall- Wallis) (Fig. 1, Figs. S4 and S5, Table S5). These results confirm the applicability of SPE for both carbon and nitrogen CSIA of atrazine, acetochlor, *S-*metolachlor, metalaxyl, butachlor, and alachlor [3,5,6,8,10,15]. The findings also extend SPE methods for CSIA to the fungicides dimethomorph and tebuconazole. Whenever larger water volumes were needed to reach the quantification limits for CSIA, water samples were divided into sub-samples ≤4 L for SPE and the extracts were recombined post-extraction. The maximum volume extracted may vary, depending on pesticide concentration, sorbent mass and matrix effect (discussed below).

Sample pre-concentration can co-enrich the target pesticides and organic compounds of the environmental matrix, leading to a so-called 'matrix effect' during GC-IRMS separation, especially for larger volumes (>4 L). Hence, field variables, such as changes in the matrix composition, pesticide concentrations and the occurrence of vegetation, should be accounted for in the matrix screening prior to sampling. For instance, the co-elution of terbutryn with the wetland water matrix did not enable carbon CSIA when water was collected in summer from the storm water wetland (data not shown). In contrast, no matrix interferences were observed for water collected in winter. Typically, compounds eluting first, such as atrazine, metalaxyl, or terbutryn, were affected stronger by the co-elution peaks.

To address the issue of co-enrichment, several clean-up procedures may be applied in the future to increase the analytical performance of pesticide CSIA extraction from environmental matrices without altering the isotope ratios of the target pesticide. These include: (i) the addition of a sorbent, such as primary secondary amine, florisil, or graphitized carbon black, etc. [1,2,18], (ii) high-performance liquid chromatography [6], and (iii) the use of molecularly imprinted polymers (MIP) [19]. MIP is likely the most effective cleanup method for CSIA. However, MIP is not commercially available for all classes of pesticides, and therefore must be specifically synthesized and validated prior to cleanup.

## Extraction from soil and sediment

### Solvent selection

**Figure 2:**
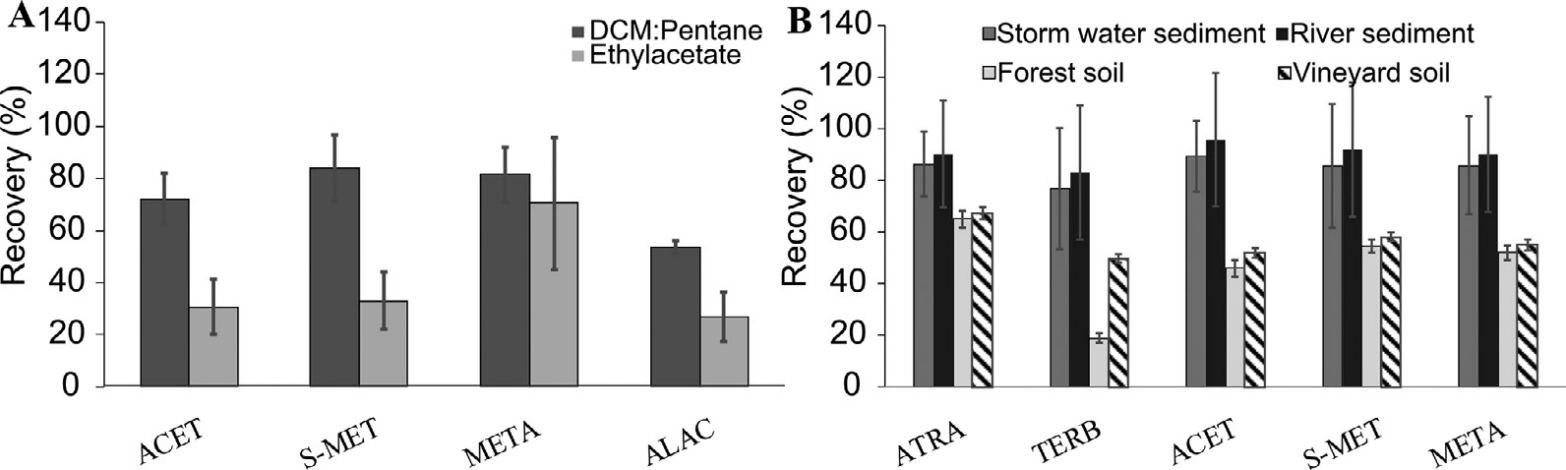
A- Recoveries of pesticide extraction from soil and sediment with DCM:Pentane and EtOAc; B- Recoveries of pesticide extraction with DCM:Pentane for different types of soil and sediment. ACET – acetochlor, S-MET – S-metolachlor, META – metalaxyl, ALAC – alachlor, ATRA– atrazine, TERB –terbutryn. Error bars denote standard deviation (1 σ, n ≥ 12).

EtOAc and DCM:Pentane (3:1) were tested as extracting solvents in an adapted MUSE method for CSIA of alachlor, acetochlor, *S-*metolachlor, and metalaxyl from soil samples. The DCM:Pentane (3:1) mix gave significantly higher recoveries than EtOAc for all compounds, except metalaxyl were both solvents yielded similar extraction recoveries (Fig. 2A). Due to its lower log *K_ow_* (1.8, Table S1), metalaxyl can also be extracted using a more polar solvent (Fig. 4), such as EtOAc. Since the method with DCM:Pentane (3:1) yielded generally higher pesticide recoveries, it was selected as extraction solvent for soil and sediments samples. Additionally, DCM:Pentane (3:1) forms separate layers with water, allowing efficient phase separation [2].

### Pesticide recoveries

The mean extraction recoveries for MUSE with DCM:Pentane (3:1) ranged from 35 ± 2 % (butachlor) up to 82 ± 17 % (dimethomorph) across the soil and sediment samples (Fig. 1, Table S5). The extraction recoveries were above 70 % for atrazine, acetochlor, *S-*metolachlor, metalaxyl, and dimethomorph. The results partly mirror the main parameters governing pesticide sorption capacities in soil/sediment matrices, including the physicochemical characteristics of the pesticide, such as the hydrophobicity, the acid dissociation constant (pKa, Table S1), and soil characteristics, including soil pH, organic matter content and surface functional groups (Table 1) [20]. The lowest extraction recovery was observed for butachlor, correlating with the highest log *K_ow_* (Table S1). The other pesticides, i.e. terbutryn, alachlor, tebuconazole, with log *K_ow_* above 3, also had an extraction recovery below 70%.

Here, we tested different pesticides with amines or amides functional groups, which do not ionize at pH 7 (Table S1). The sorption mechanism governing the action of nonionic pesticides includes physical interaction, e.g. the hydrophobic effect, when a nonpolar compound interacts with a nonpolar soil organic component, or chemical interactions, e.g. hydrogen bonding, interactions with humic substances and clay in the soil [20]. Hydrophobicity (i.e., *K_ow_*) and soil sorption properties (i.e., *K_oc_*, adsorption coefficient of soil) are thus prevailing variables influencing the extraction recovery of pesticides.

In addition, the physicochemical characteristics of the soil or the sediment affect the extraction efficiency. Fig. 2B shows the variation of extraction efficiencies among environmental samples. For all compounds, extraction recoveries were above 70% in sediment samples (Fig. 2B). In contrast, the forest soil with the highest organic carbon (OC) content (Table 1), displayed the lowest extraction recovery. Interestingly, the forest soil was characterized by a pH=3.4. The low soil pH may increase the fraction of protonated functional groups in soil, such as carboxylic or thiol, thereby enhancing sorption of nonionic pesticides [21]. The low soil pH may also enhance ionic interactions between soil components and pesticides, such as terbutryn, with a pKa value of 4.3, which may reduce extraction recovery (Fig. 2B, Table S1).

The vineyard soil (Rouffach, France) featured low initial water content (13 ± 7%) and lower extraction yields (Fig. 2B), although the OC contents and pH of the vineyard soil, and both the river and wetland sediments (Rouffach and Alteckendorf) were similar (Table 1). The fraction of non-extractable residues (NER) may increase with lower water content due to the more hydrophobic nature of soil organic matter controlling pesticide extraction [22]. Therefore, together with changes in soil texture and structure, pH, soil OC content and the initial soil water content appear as fundamental parameters controlling pesticide aging in soil [23] and thus extraction from soil and sediment samples.

In addition, seasonal variations of soil and sediment properties may affect the extraction recovery. For instance, the extraction recovery of dimethomorph from storm water sediment was lower in summer than in winter (Rouffach, France), which may be due to higher OC content of sediment and/or different quality of OC in the sediment in summer (Fig. S6). In addition, only partial mixing of the solvent (DCM:Pentane) and storm water sediment sampled in summer was observed. This may explain the decrease by three-fold of the dimethomorph recovery from the storm water sediment (Fig. S7). In this case, a more polar extraction solvent, such as MeOH, may be used in combination with a CaCl_2_ extraction step to replace organic compounds sorbed to negatively charged organic matter with cations [24]. A two-step extraction method for the storm water sediment collected in summer and the MUSE extraction method used for storm water sediment collected in winter both yielded recoveries of about 80% (Fig. S7). In this method, tailoring extraction solvent to the soil type (i.e., use of CaCl_2_ and MeOH), increased the extraction recovery (Fig. 4). Extraction recoveries were similar to those with ASE extraction of dimethomorph from two types of sediment from California, using DCM as extraction solvent [25].

### Effect on stable isotope signatures

**Figure 3:**
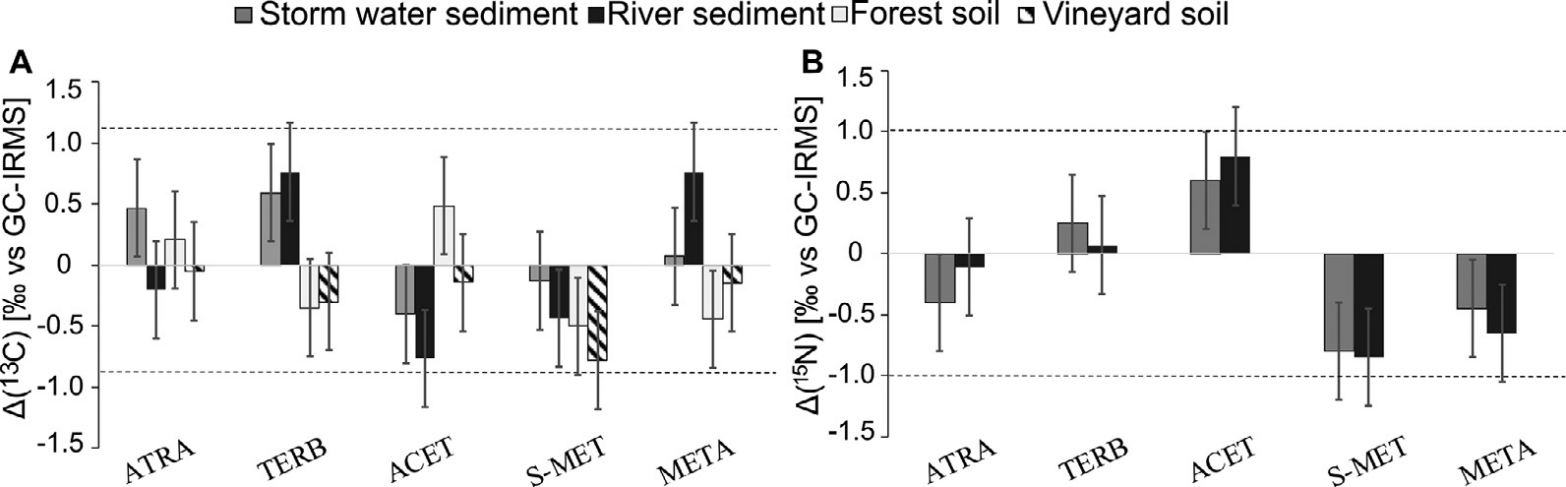
Effect of the MUSE extraction method on A – Carbon isotope values (Δ(^13^C) _[‰ vs GC-IRMS]_) for the different types of soils and sediments and B – Nitrogen isotope values (Δ(^15^N) _[‰ vs GC-IRMS]_) for the different types of soil and sediment. Dashed lines represent ±1‰ (significance threshold) from the measured isotope values of in-house standards. ATRA– atrazine, TERB –terbutryn, ACET - acetochlor, S-MET - S-metolachlor, META – metalaxyl. Error bars denote standard deviation for all concentrations.

Carbon and nitrogen stable isotope composition of pesticides were not significantly affected (<0.5‰ for Δ(^13^C) and <0.8‰ for Δ(^15^N)) by the soil type or the pesticide concentrations (Fig. 1, Fig. 3). This is in line with the literature for soil extraction using the MUSE method for both carbon and nitrogen CSIA of atrazine, acetochlor,*S-*metolachlor, metalaxyl, butachlor, and alachlor [[9], [10], [11],^15^]. This study is, to the best of our knowledge, the first report of soil extraction of dimethomorph and tebuconazole for carbon and nitrogen CSIA.

**Figure 4:**
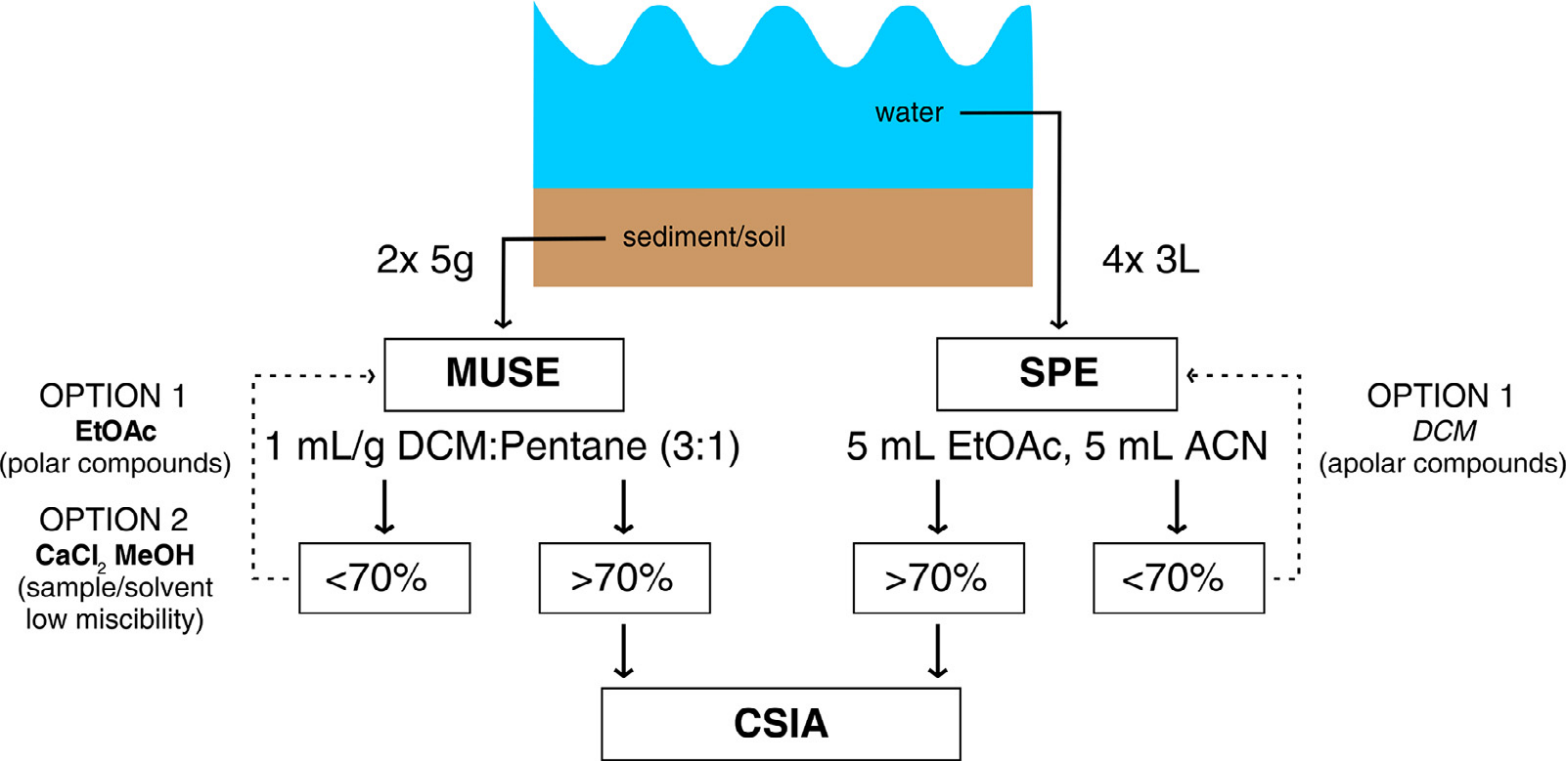
Pesticide extraction from sediment/soil and water samples for CSIA. Possible solvent alternatives (suggested – italicized, tested in this study– bolded) are mentioned when extraction recovery is <70%. MUSE - modified ultrasonic-assisted extraction, SPE - solid-phase extraction, CSIA - compound-specific isotope analysis

Overall, these results highlight that pesticide extraction from soil samples for CSIA requires considering several practical aspects. First, pre-concentration of soil extracts may increase the chromatogram baseline during GC-IRMS measurements, especially for *δ*^13^C analysis (Fig. S9). This may severely compromise the analytical performance (higher MDL and lower accuracy), even when co-elution is limited. The application of MIP with high specificity in the pre-concentration step may significantly reduce the matrix effect of soil compounds and thus reduce the MDL. Finally, injection of high carbon loads, especially during *δ*^15^N measurements, may significantly increase the maintenance of IRMS system. This may imply frequent oven and column replacement, and capillaries blockage.

## Extraction from plants

### Extraction recovery and effect on stable isotope signatures

The mean extraction recovery for plant material ranged from 40 ± 16 % (Dimethomorph) up to 59 ± 10 % (*S* - metolachlor) (Fig. 1, Table S5). Extraction recoveries were slightly higher for the roots than for the aerial parts (data not shown). Overall, extraction recoveries from plant were lower than those for soil and sediment, suggesting that pesticide sorption in the plant matrix was higher. However, extraction efficiencies were similar to those reported for ACE extraction for HCHs from plants [12]. Despite low extraction efficiencies, *δ^1^*^3^C of *δ*^15^N of pesticides were not affected by the extraction from plant material (Fig. 1, Table S5).

In the future, other extraction methods of pesticides from plants, such as solid-phase microextraction, QuEChERS, superfluid extraction, and Soxhlet extraction, routinely tested for concentration analysis, could also be evaluated for CSIA in order to increase the recovery [26,27].

## Implication for ME-CSIA from environmental matrices

The applicability of extraction methods for ME-CSIA to environmental samples is an essential step. The tested SPE methods allowed carbon CSIA from water samples for all studied pesticides at concentrations above 100 to 200 ng/L (Table S6). In contrast, nitrogen CSIA required several µg/L of pesticides in water. In a previous study, concentrations of *S-*metolachlor and acetochlor in water ranged from <100 to 66100 ng/L at the plot or at the outlet of the agricultural catchment [28]. In urban catchments, biocides used in paints and renders, such as terbutryn, may leach from the facade materials and result in heavily polluted runoff (up to several mg/L biocides) [29]. Hence, carbon CSIA of pesticides and biocides appears feasible from agricultural samples across the agricultural season, and from most leachates from construction material in urban settings. For some compounds, the SPE method for carbon CSIA can also be used for nonpoint sources of pollution, although wider application would require reaching levels of pg/L to several ng/L. In contrast, nitrogen CSIA may be restricted to areas close to the source, following application, and/or during runoff events (Table S6).

The tested extraction method enabled carbon CSIA from soil/sediment and plant samples with pesticide concentrations ranging from 300 to 1000 ng/g and 300 to 500 ng/g, respectively (Table S6). Although *S-*metolachlor concentrations in soil can reach 8 µg/g following application [11], pesticide concentrations typically range from pg/g to several ng/g in agricultural soils [30]. This currently restricts pesticide CSIA to source areas, following application. Furthermore, nitrogen CSIA in soils and plants would typically require more than 10 µg/g, currently restricting its application to laboratory studies. Developing simple and free of isotope-effect clean-up methods for environmental matrices and pesticides might reduce the current concentration ranges of ME-CSIA. Altogether, this study illustrates the diversity of candidate compounds and environmental matrices for ME-CSIA and the current potential and limitations of pesticide extraction methods, paving the way for the wider use of ME-CSIA to assess pesticide degradation in environmental compartments.

## Declaration of Competing Interest

The authors declare that they have no known competing financial interests or personal relationships that could have appeared to influence the work reported in this paper.

## Data Availability

Data will be made available on request.

## Appendix Supplementary materials

Image, application 1
