# Supplementary material for: Simple extraction methods for pesticide compound-specific isotope analysis from environmental samples
Source: MethodsX. 2022 Oct 13;9:101880. doi: 10.1016/j.mex.2022.101880 (PMC9597100; doi:10.1016/j.mex.2022.101880)
Supplement: Supplementary file 1 [file mmc1.docx]

Supporting Information

**Simple extraction methods for pesticide compound-specific isotope analysis from environmental samples**

Tetyana Gilevska^1^, Charline Wiegert^1^, Boris Droz^1^, Tobias Junginger^1^, Maria Prieto- Espinoza^1^, Adrien Borreca^1^, Gwenaël Imfeld^1^

^1^ Université de Strasbourg, CNRS/ENGEES, ITES UMR 7063, Institut Terre et Environnement de Strasbourg, 5 Rue René Descartes, 67000 Strasbourg, France

Table S1. Chemical properties of the studied compounds. ^b^ - banned as pesticides but used as paint additives, NA – not applicable, - not measured.

| Common name | Use | Approval in EU (revision date)  http://sitem.herts.ac.uk/aeru/ | Structure | Chemical family | Chemical formula | Half-life in Water-sediment (days)  http://sitem.herts.ac.uk/aeru/ | Solubility in water (20 ºC, mg L^-1^)  http://sitem.herts.ac.uk/aeru/ | Octanol/water coefficient (Log*Kow*) ; pH7, 20°C) | pKa, 25°C | Sorption capacities, ${logK}_{oc}$ | |
| --- | --- | --- | --- | --- | --- | --- | --- | --- | --- | --- | --- |
|  |  |  |  |  |  |  |  |  |  | Experimental, Rouffach sediment  [1] | Predicted consensus from EPA CompTox [2] |
| atrazine | herbicides | banned (2004) | 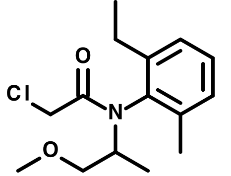 | Triazine | $C_{8}H_{14}ClN_{5}$ | 80 | 35 | 2.7 | 1.7 | 1.5 – 2.6 | 2.1 – 2.2 |
| terbutryn |  | banned (2002)^b^ | 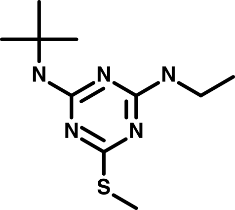 |  | $C_{10}H_{19}N_{5}S$ | 60 | 25 | 3.7 | 4.3 | 2.8 – 3.1 | 2.8 – 2.9 |
| acetochlor |  | banned (2013) | 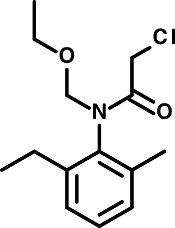 | Chloroacetamide | $C_{14}H_{20}ClNO_{2}$ | 20 | 280 | 4.1 | NA | 2.2 – 2.5 | 2.3 – 2.5 |
| alachlor |  | banned (2009) | 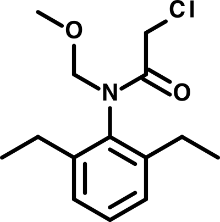 |  | $C_{14}H_{20}ClNO_{2}$ | 2 | 240 | 3.1 | NA | - | 2.3 – 2.7 |
| butachlor |  | banned (2009) | 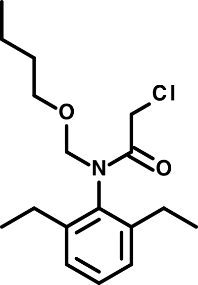 |  | $C_{17}H_{26}ClNO_{2}$ | 200 | 20 | 4.5 | NA | - | 2.9 |
| *S*-metolachlor |  | approved (2019) | 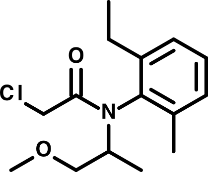 |  | $C_{15}H_{22}ClNO_{2}$ | 47 | 480 | 3 | NA | 2.3 – 2.6 | 2.4 – 2.5 |
| dimethomorph | fungicide | approved (2022) | 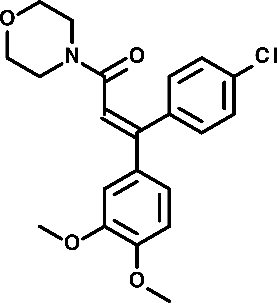 | Morpholine | $C_{21}H_{22}ClNO_{4}$ | 38 | 29 | 2.7 | -1.3 | - | 2.4 – 3.4 |
| tebuconazole |  | approved (2022) | 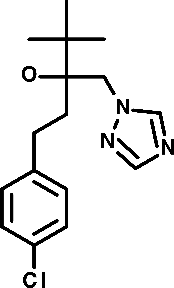 |  | $C_{16}H_{22}ClN_{3}O$ | 365 | 36 | 3.7 | 5.0 | - | 2.6 – 3.0 |
| metalaxyl |  | approved (2023) | 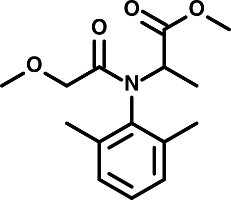 | Phenylamide | $C_{15}H_{21}NO_{4}$ | 32 | 8400 | 1.8 | 1.4 [3] | 1.1 – 1.7 | 1.6 – 1.7 |

**S1. Pesticide extraction from water samples**

The extraction procedure was performed with an AutoTrace 280 solid-phase extraction (SPE) system (Dionex®, CA, USA) for the simultaneous extraction of 6 samples. Maximum volume of water that can be pumped this system is 4 L. SPE cartridges, SolEx C18 cartridges (1 g, Dionex®, CA, USA) were washed with 5 mL of EtOAc, followed by 5 mL of ACN. The cartridges were then sequentially conditioned by 10 mL of ultrapure water. Cartridges were loaded with the samples and dried under nitrogen flux for 10 min. Elution of pesticides was performed by 5 mL of EtOAc followed by 5 mL of ACN. The extract was subsequently concentrated under nitrogen flux to 1 droplet and suspended in 0.5 mL of ACN for quantification and isotope analyses. For the isotope analysis, lower concentration samples were further pre-concentrated up to 10 times, by placing 250 µL of the sample in the glass GC vial inserts, followed by evaporation under nitrogen flux the samples and subsequent reconstitution in 0.5 mL.

Table S2. Hydrochemistry of the environmental waters. Analytical uncertainties were 5% for major ions, metals and carbon concentrations. Precision was ± 0.5% for conductivity and dissolved oxygen measurements, ± 0.01 unit for pH and ± 10 mV for redox potential. D.O. – dissolved oxygen, TOC – total organic carbon, DOC – dissolved organic carbon.

|  |  | Vineyard runoff water, Rouffach, France | Crop runoff water, Alteckendorf, France | River water, Souffel, France |
| --- | --- | --- | --- | --- |
|  |  | 47°57'43'' N, 7°17'26'' E | 48°47'17"N, 7°35'25"E | 48°38'20"N, 7°44'35"E |
| Compound | Unit | Range | | |
| D.O. | mg/L | 0.2 – 3.9 | - | - |
| Redox | mV | - 44 – 47 | - | 123 – 128 |
| pH | - | 7.0 –7.3 | 6.0 –7.8 | 7.0 – 7.3 |
| E.C. | µS/cm | 448 – 925 | - | 900 – 1100 |
| TOC | mg/L | 7.6 – 12.4 | 0.4 – 1.9 | 4.12 |
| DOC | mg/L | 6.9 – 12.4 | 0.2 – 50 | 3.19 |
| TSS | mg/L | 2 – 172 | 3 – 65 | - |
| Al^3+^ | mg/L | 0.04 – 0.2 | 4 – 50 | - |
| Na^+^ | mg/L | 3.3 – 12.5 | 8.2 | 13.5 – 14.2 |
| Mg^2+^ | mg/L | 3.3 – 12.3 | 23.3 | 45.9 – 47.3 |
| K^+^ | mg/L | 3.4 – 7.1 | 0.8 | 6.4 – 14.0 |
| Fe ^3+^ | mg/L | 0.2 – 2.8 | - | 13.5 – 14.2 |
| Fe^2+^ | mg/L | 0.8 – 3.3 | - | - |
| Cu^2+^ | mg/L | 3.8 – 37 | - | - |
| SO_4_ ^2-^ | mg/L | 10.5 – 48.5 | 31.8 | 213.4 |
| NO_2_^-^ | mg/L | n.d. – 2.7 | 0.1 – 0.8 |  |
| NO_3_^-^ | mg/L | n.d. – 2.7 | 27 – 114 | 62.8 – 63.9 |
| NH_4_^+^ | mg/L | n.d. – 3.2 | 1 – 12 | n.d. – 0.3 |
| PO_4_^3-^ | mg/L | 0.4 – 0.6 | 0.4 – 1.9 | - |
| Cl^-^ | mg/L | 5.0 – 22.4 | 41.69 | 34.4 – 50.9 |

Table S3. Physicochemical properties of the selected soils.

|  |  | Forest soil, Strengbach, France | Vineyard soil, Rouffach, France | River sediment  Alteckendorf, France | Storm water sediment  Rouffach, France, |
| --- | --- | --- | --- | --- | --- |
|  |  | 48°12'58"N,7°11'53"E | 47°57'44", 7°17'27"E | 48°47'17"N, 7°35'25"E | 47°57'43'' N, 7°17'26'' E |
| **Texture** | Clay (< 2µm) [%] | 7 ± 0.3 | 23 ± 2 | 14 ± 0.4 | 24 ± 8 |
|  | Silt (2 - 50 µm) [%] | 32 ± 1 | 68 ± 9 | 73 ± 2 | 45 ± 19 |
|  | Sand (50 - 2000 µm) [%] | 62 ± 2 | 9 ± 7 | 13± 0.4 | 32 ± 22 |
|  | Organic carbon (O_C_) [%] | 4.5 ± 0.2 | 1.1 ± 0.2 | 2.0 ± 0.2 | 2.5± 0.4 |
|  | pH | 3.4 ± 0.1 | 7.9 ± 0.1 | 7.8 ± 0.1 | 7.6± 0.1 |
|  | CaCO_3_ [%] | - | 27 | 15 | 19 ± 7 |
|  | Cation exchange capacity (CEC) [cmol^+^/kg] | 14 | 18 | 15 | 14 |
|  | Bulk Density [g/cm^3^] | 1.0 | 1.5 | - | 1.7 |
|  | Water content [%] | 47 ± 12 | 13 ± 7 | 39 ± 3 | 41 ± 6 |
| **Major elements [% dry soil] (ICP-AES analysis)** | SiO_2_ | 61.3 | 52.7 | 65 | 48 |
|  | Al_2_O_3_ | 14.9 | 7.7 | 8.5 | 11 |
|  | MgO | 0.6 | 1.4 | 1.4 | 2.0 |
|  | CaO | 0.0 | 15.1 | 6.7 | 13 |
|  | Fe_2_O_3_ | 2.6 | 3.2 | 3. | 4.4 |
|  | MnO | 0 | 0.1 | 0.1 | 0.1 |
|  | TiO_2_ | 0 | 0.5 | 0.7 | 0.6 |
|  | Na_2_O | 0.6 | 0.8 | 1.0 | 0.5 |
|  | K_2_O | 4.3 | 1.8 | 1.9 | 2.3 |
|  | P_2_O_5_ | 0.2 | 0.4 | 0.2 | 0.3 |

Table S4. Information of standards quantification and carbon and nitrogen stable isotope fractionation, mean value ± SD, n- number of samples.

| Compound | Ions used for quantification  (GC-MS) | δ^13^C (‰)  (EA-IRMS)  n =3 | δ^15^N (‰)  (EA-IRMS)  n =3 | δ^13^C (‰)  (GC-IRMS)  n = 30 | δ^15^N (‰)  (GC-IRMS)  n = 30 |
| --- | --- | --- | --- | --- | --- |
| Atrazine | 215/200 | -25.7±0.2 | -2.2±0.2 | -25.7±0.4 | -1.8±0.5 |
| Terbutryn | 226/185 | -28.8±0.1 | -2.9±0.2 | -29.0±0.5 | -3.0±0.3 |
| Acetochlor | 142/162 | -29.1±0.1 | -2.4±0.2 | -29.5±0.4 | -2.9±0.4 |
| Alachlor | 188/162 | -29.4±0.1 | -2.5±0.2 | -29.5±0.5 | -2.3±0.4 |
| Butachlor | 176/160 | -26.6±0.1 | 0.6±0.2 | -27.7±0.5 | 0.6±0.5 |
| Metalaxyl | 206/132 | -31.5±0.1 | 0.3±0.2 | -32.3±0.5 | 0.1±0.4 |
| *S-*Metolachlor | 215/200 | -30.5±0.1 | 0.4 ±0.2 | -31.0±0.5 | 0.3 ±0.3 |
| Tebuconazole | 249/173 | -29.3±0.1 | 1.3±0.2 | -29.9±0.4 | 1.3±0.4 |
| Dimethomorph Z | 301/165 | -33.2±0.2 | 0.8±0.2 | -34.2±0.5 | - |
| Dimethomorph E | 301/165 | -33.2±0.2 | 0.8±0.2 | -33.8±0.5 | - |

Figure S1. Recovery of the studied pesticides from 1L and varying concentrations.


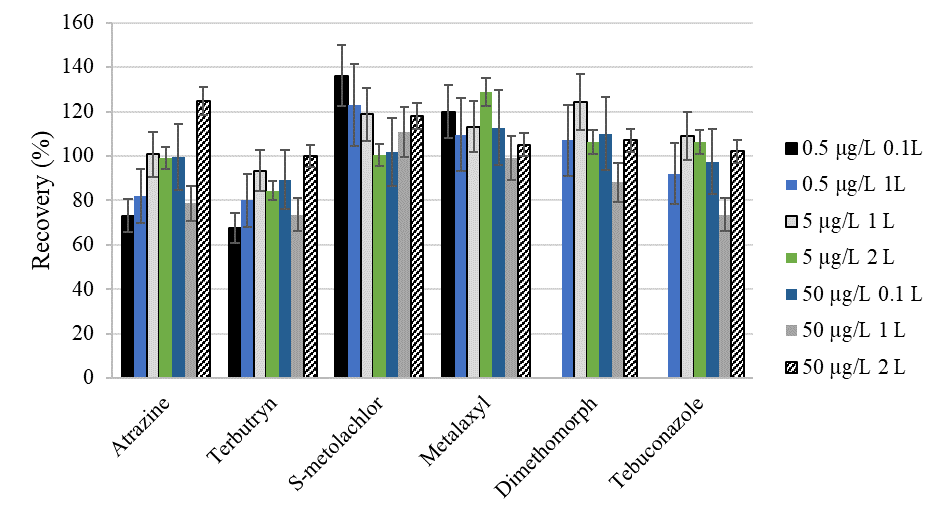


Figure S2. Recovery for the studied pesticides for different water volumes and concentrations.

Figure S3. Recovery for the studied pesticides for different matrixes and volumes.


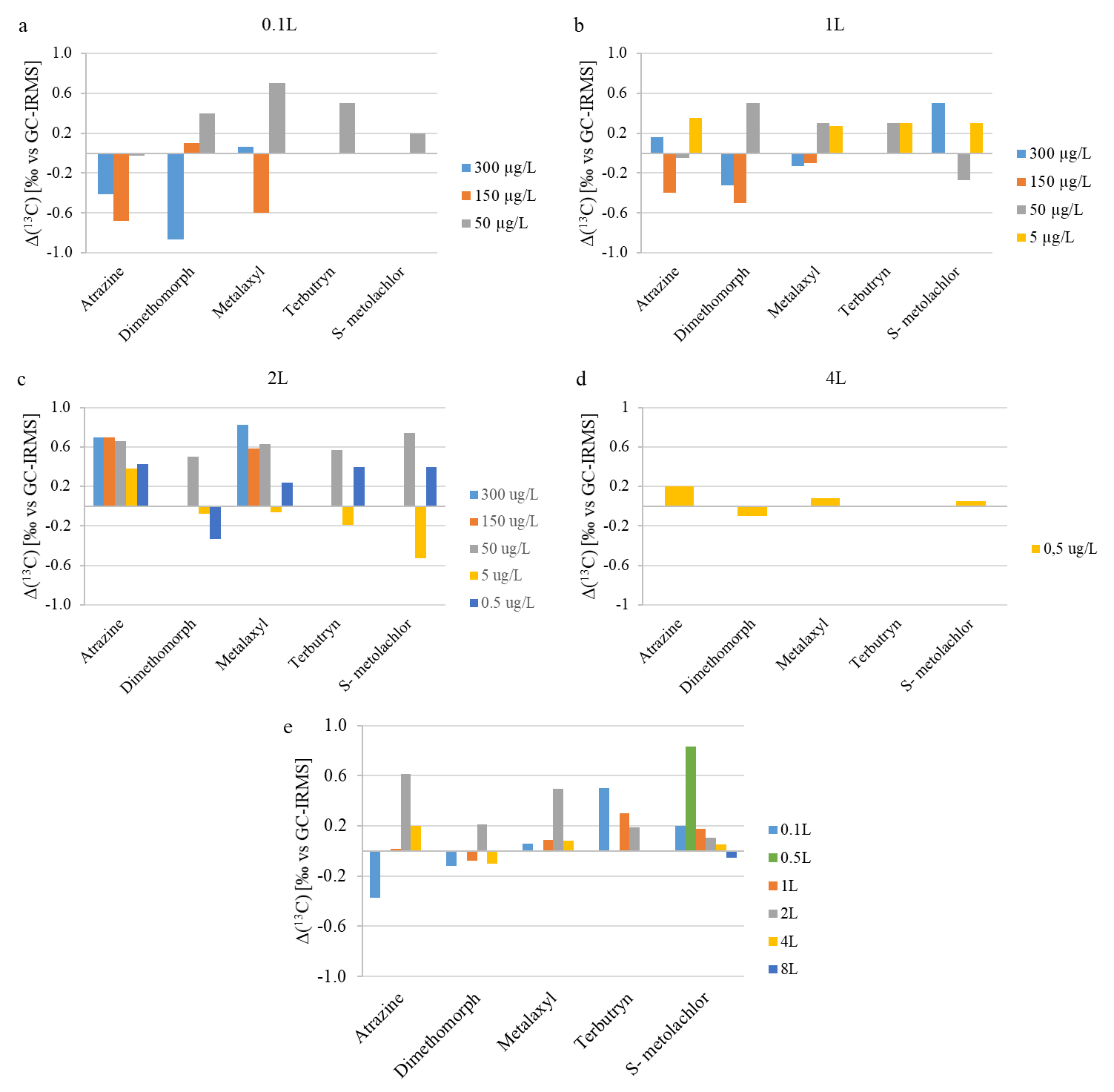


Figure S4. Effect of extraction on carbon isotope signatures of standard (Δ(^13^C) _[‰ vs GC-IRMS]_) for different concentrations and volumes (a,b,c,d) and among volumes (all concentrations) (e).


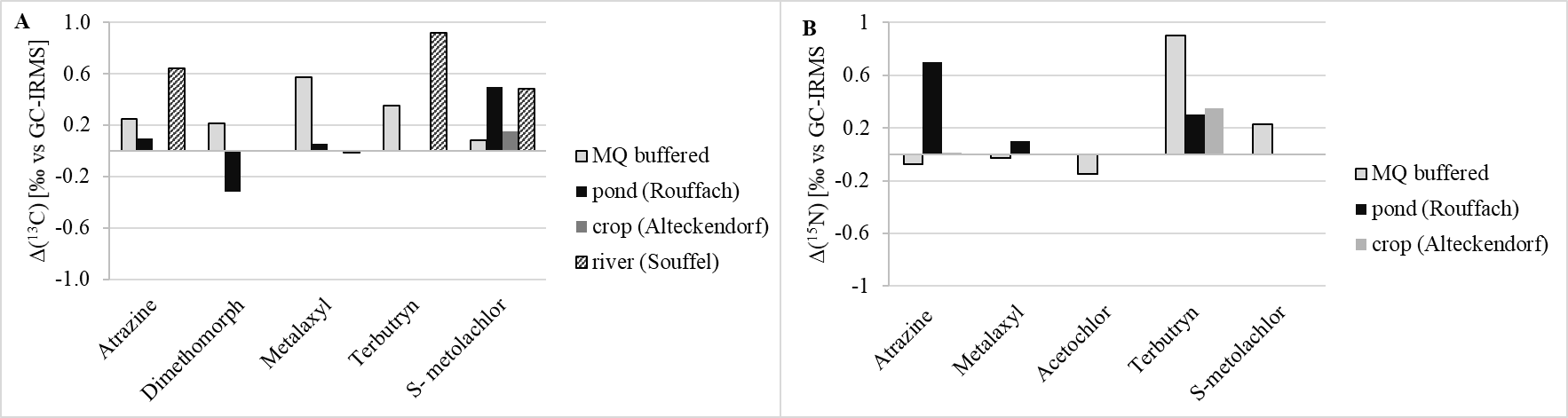


Figure S5. Effect of the SPE extraction method and different water matrices on A - Δ(^13^C), carbon isotope signatures, and B - Δ(^15^N), nitrogen isotope signatures.


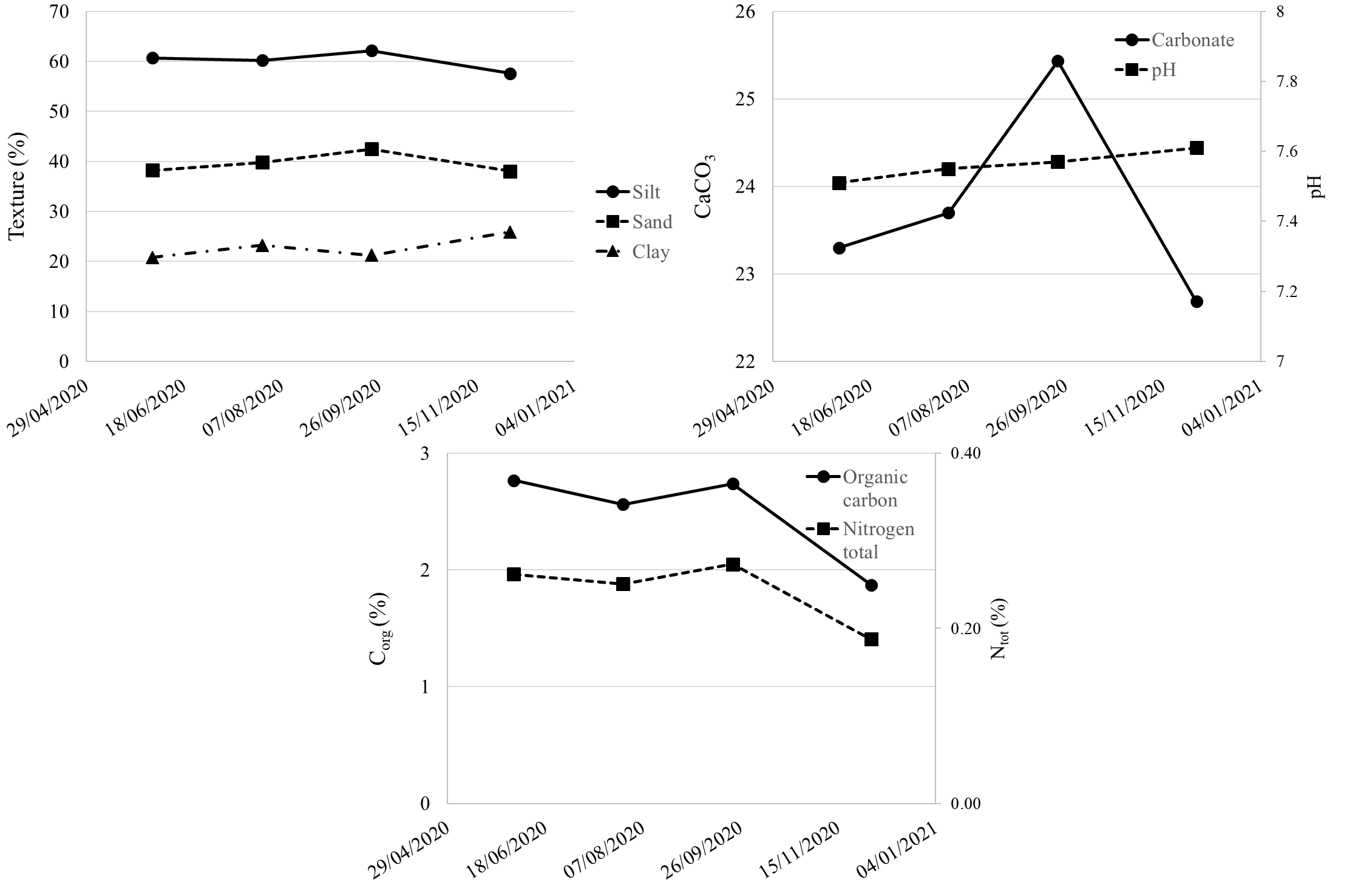


Figure S6. Properties of the storm water Rouffach sediment over time (from June to December).


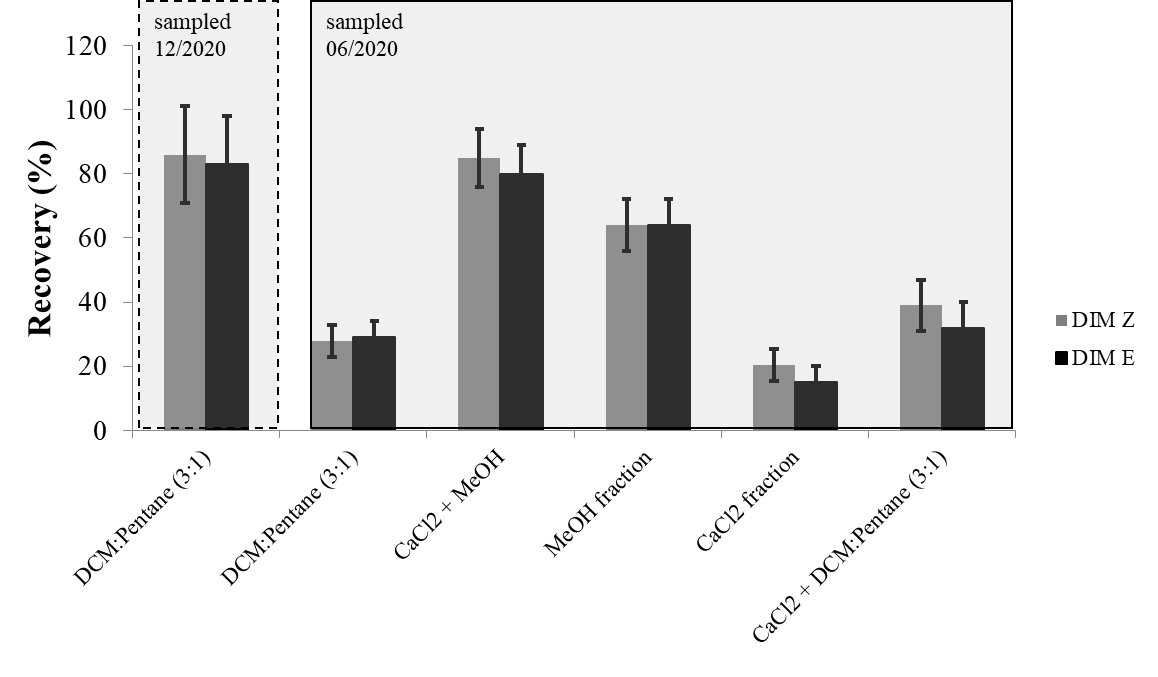


Figure S7. Recovery of Z and E isomers of dimethomorph with a DCM:Pentane extraction (3:1) and a 2-step extraction with CaCl_2_ and MeOH. Shading highlights sampled collected in winter period and in summer period.


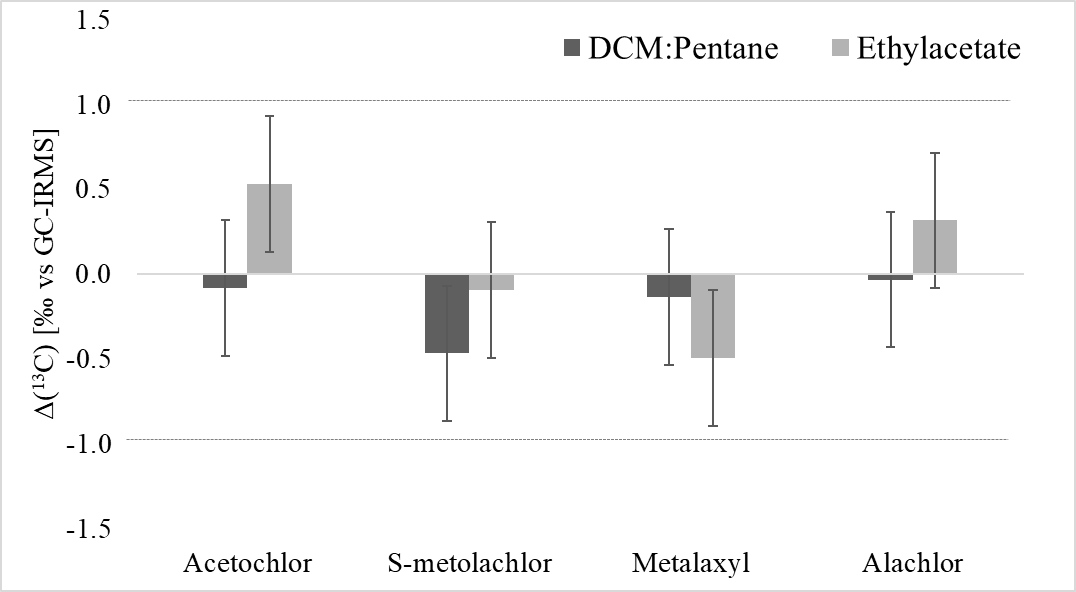


Figure S8. Effect of MUSE extraction on carbon isotope values of sediment and soil isotope fractionation of pesticides for different extraction solvent.


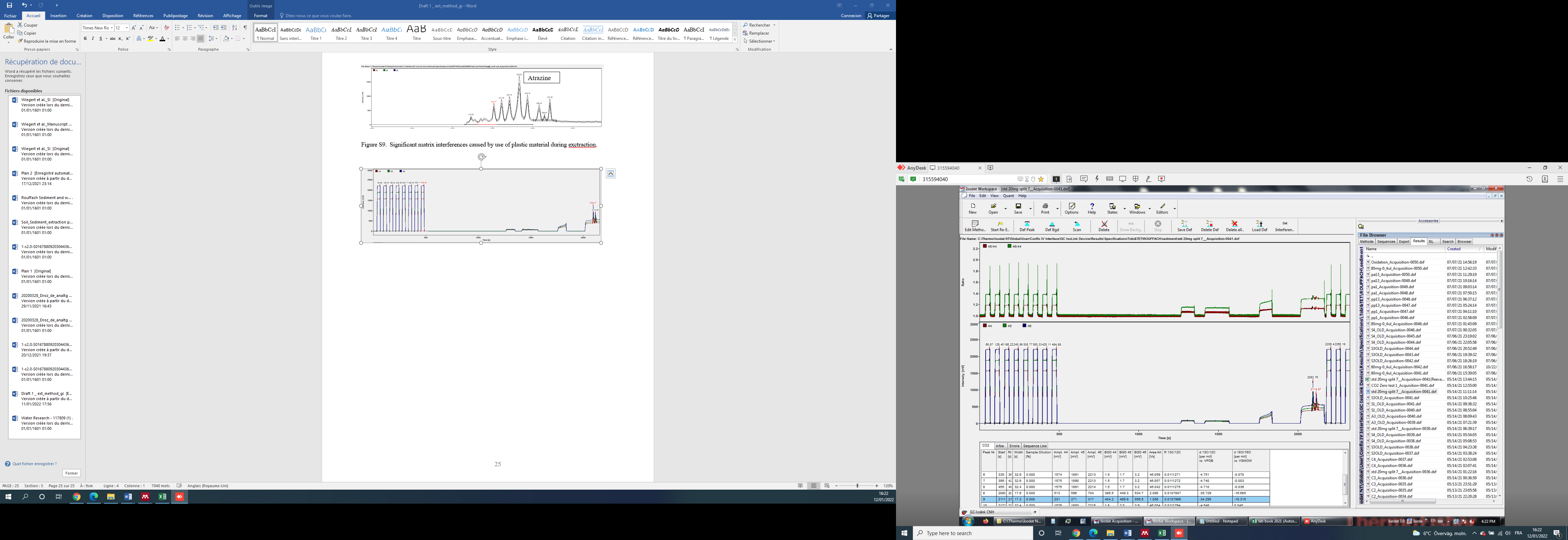


1. Sample extracted with DCM:Pentane


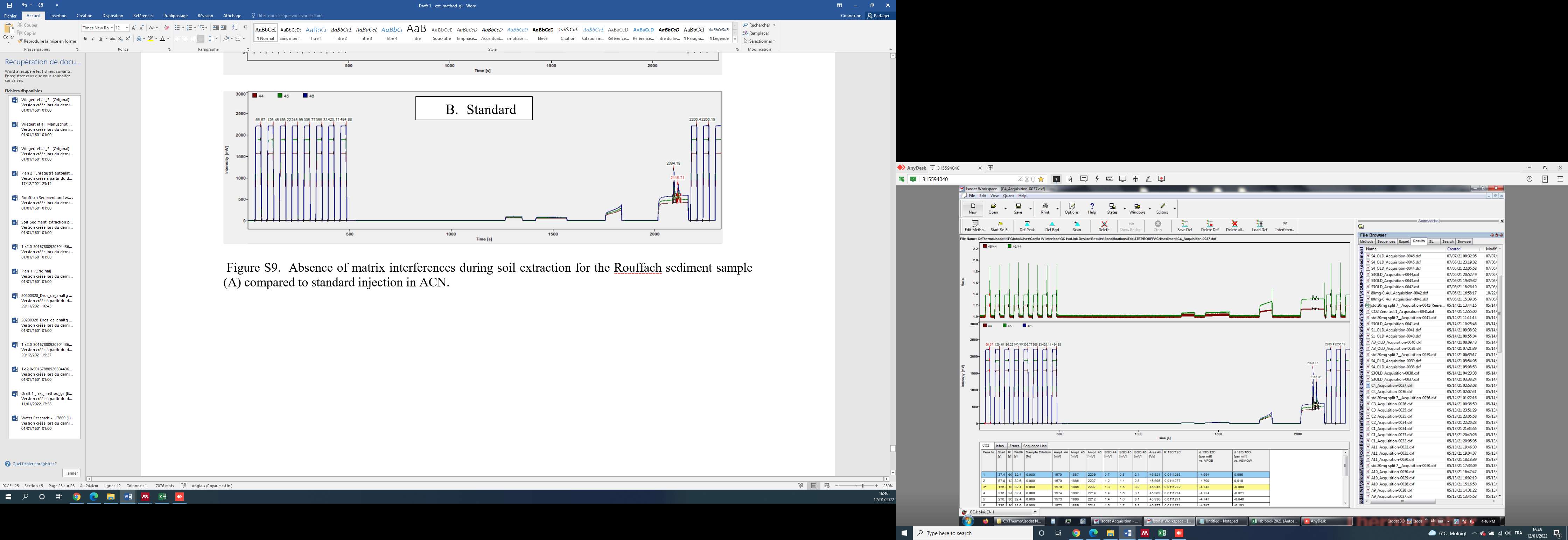


1. Sample extracted with MeOH


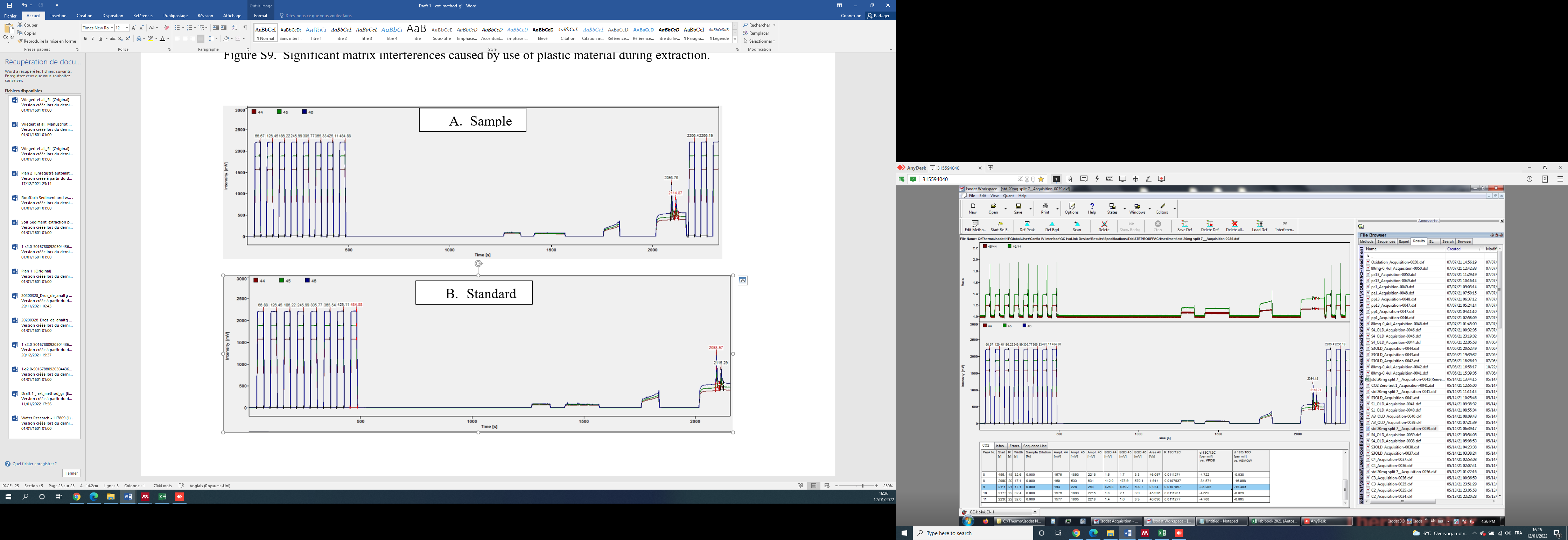


1. Standard in ACN

Figure S9. Chromatogram of dimethomoph (isomer Z and E) with no significant matrix interferences during soil extraction of the Rouffach sediment sample (A and B) compared to standard injection in ACN (C).

Table S5. Extraction recovery, isotope effect (Δ(^13^C), Δ(^15^N)) for SPE, soil/sediment, and plant extraction. (n) - the number of samples extracted, NA – not analysed.

|  |  |  | Atrazine | Terbutryn | Acetochlor | *S-*metolachlor | Metalaxyl | Dimethomorph | Tebuconazole | Butachlor | Alachlor |
| --- | --- | --- | --- | --- | --- | --- | --- | --- | --- | --- | --- |
| **SPE** | Recovery | % ± SD | 87 ± 8 (41) | 88 ± 10 (31) | 102 ± 6 (44) | 115 ± 12 (73) | 95 ± 8 (61) | 107 ± 24 (12) | 92 ± 20 (16) | 77 ± 6 (30) | 98 ± 5 (39) |
|  | Δ(^13^C), GC-IRMS | ‰ ± SD | -0.01 ± 0.7 (87) | 0.2 ± 0.7 (50) | -0.2 ± 0.3 (44) | 0.03 ± 0.4 (94) | -0.04 ± 0.6 (106) | -0.3 ± 0.8 (39) | 0.01 ± 0.6 (35) | 0.5 ± 0.2 (30) | 0.02 ± 0.2 (56) |
|  | Δ(^13^C), EA-IRMS | ‰ ± SD | -0.1 ± 0.7 (87) | 0.6 ± 0.7 (50) | -0.6 ± 0.3 (44) | -0.1 ± 0.4 (94) | -0.3 ± 0.6 (106) | 0.5 ± 0.8 (39) | -0.5 ± 0.6 (35) | -0.1 ± 0.3 (30) | -0.2 ± 0.1 (56) |
|  | Δ(^15^N), GC-IRMS | ‰ ± SD | 0.3 ± 0.7 (21) | 0.6 ± 0.6 (17) | 0.6 ± 0.6 (17) | -0.2 ± 0.5 (8) | 0.3 ± 0.8 (7) | NA | 0.0 ± 0.6 (21) | 0.1 ± 0.7 (10) | -0.1 ± 0.2 (4) |
|  | Δ(^15^N), EA-IRMS | ‰ ± SD | 0.3 ± 0.7 (21) | 0.9 ± 0.6 (21) | 0.2 ± 0.6 (17) | 0.6 ± 0.5 (8) | 0.1 ± 0.8 (7) | NA | 0.0 ± 0.6 (21) | 0.3 ± 0.7 (10) | 0.1 ± 0.2 (4) |
| **Sediment/soil** | Recovery | % ± SD | 77 ± 12 (237) | 57 ± 14 (216) | 71 ± 10 (240) | 72 ± 13 (240) | 71 ± 11 (240) | 82 ± 17 (25) | 66 ± 14 (21) | 35 ± 2 (24) | 54 ± 9 (24) |
|  | Δ(^13^C), GC-IRMS | ‰ ± SD | 0.04 ± 0.4 (202) | 0.1 ± 0.3 (202) | -0.1 ± 0.2(218) | -0.5 ± 0.3 (218) | -0.1 ± 0.4 (218) | -0.2 ± 0.6 (12) | 0.4 ± 0.2 (8) | -0.9 ± 0.1 (114) | 0.1 ± 0.2 (114) |
|  | Δ(^13^C), EA-IRMS | ‰ ± SD | -0.5 ± 0.4 (202) | 0.1 ± 0.2 (202) | 0.3 ± 0.2 (218) | -0.5 ± 0.3 (218) | -0.4 ± 0.4 (218) | 0.6 ± 0.6 (12) | 0.4 ± 0.2 (8) | -1.7 ± 0.1 (114) | 0.4 ± 0.2 (114) |
|  | Δ(^15^N), GC-IRMS | ‰ ± SD | -0.2 ± 0.5 (25) | 0.3 ± 0.5 (25) | 0.5 ± 0.1 (15) | -0.9 ± 0.3 (15) | -0.3 ± 0.6 (28) | NA | 0.4 ± 1.0 (10) | NA | NA |
|  | Δ(^15^N), EA-IRMS | ‰ ± SD | -0.6 ± 0.5 (25) | 0.2 ± 0.5 (25) | 0.9 ± 0.1 (15) | -1.0 ± 0.3 (15) | -0.2 ± 0.6 (28) | NA | 0.4 ± 1.0 (10) | NA | NA |
| **Plant** | Recovery | % ± SD | 54 ± 7 (30) | 49 ± 12 (30) | NA | 59 ± 10 (30) | 53 ± 11 (30) | 40 ± 16 (30) | 50 ± 14 (30) | NA | NA |
|  | Δ(^13^C), GC-IRMS | ‰ ± SD | -0.7 ± 0.6 (8) | -0.2 ± 0.5 (12) | NA | -0.3 ± 0.2 (12) | -0.2 ± 0.5 (12) | -0.4 ± 0.4 (12) | 0.0 ± 0.2 (12) | NA | NA |
|  | Δ(^13^C), EA-IRMS | ‰ ± SD | -0.6 ± 0.6 (8) | 0.2 ± 0.5 (12) | NA | -0.9 ± 0.2 (12) | -0.5 ± 0.5 (12) | 1.8 ± 0.4 (12) | -0.3 ± 0.3 (12) | NA | NA |
|  | Δ(^15^N), GC-IRMS | ‰ ± SD | -0.7 ± 0.3 (8) | 0.0 ± 0.5 (8) | NA | -0.4 ± 0.4 (8) | 0.8 ± 0.3 (8) | NA | 0.3 ± 0.3 (8) | NA | NA |
|  | Δ(^15^N), EA-IRMS | ‰ ± SD | 0.7 ± 0.3 (8) | 0.3± 0.5 (8) | NA | -0.3 ± 0.4 (8) | 0.9 ± 0.3 (8) | NA | 0.4 ± 0.3 (8) | NA | NA |

Table S6. Method detection limit (MDL) for δ^13^C and δ^15^N compound specific isotope analysis of pesticides for SPE, soil/sediment, and plant extraction. NA – not analysed.

|  |  |  | Atrazine | Terbutryn | Acetochlor | *S-*metolachlor | Metalaxyl | Dimethomorph | Tebuconazole | Butachlor | Alachlor |
| --- | --- | --- | --- | --- | --- | --- | --- | --- | --- | --- | --- |
| MDL δ^13^C | On column | ng of C | 6 | 7 | 6 | 7 | 5 | 5 | 6 | 7 | 6 |
|  | In extract | mg/L | 4 | 5 | 3 | 4 | 3 | 4 | 4 | 4 | 3 |
|  | In water 12L (3*4L) | µg/L | 0.2 | 0.2 | 0.1 | 0.1 | 0.1 | 0.2 | 0.2 | 0.2 | 0.1 |
|  | In soil/sediment (10 g) | µg/g | 0.5 | 0.7 | 0.4 | 0.4 | 0.3 | 0.4 | 0.5 | 1.1 | 0.6 |
|  | In plant (5 g) | µg/g | 0.4 | 0.5 | NA | 0.3 | 0.3 | 0.4 | 0.4 | NA | NA |
| MDL δ^15^N | On column | ng of N | 37 | 87 | 17 | 17 | 18 | NA | 100 | 21 | 37 |
|  | In extract | mg/L | 38 | 100 | 111 | 113 | 122 | NA | 100 | 157 | 38 |
|  | In water 12 L (3*4 L) | µg/L | 2.0 | 3.9 | 4.5 | 3.7 | 5.6 | NA | 4.5 | 8.5 | NA |
|  | In soil/sediment (10 g) | µg/g | 4.8 | 14.5 | 15.4 | 13.4 | 15.0 | NA | 13.9 | 44.9 | NA |
|  | In plant (5 g) | µg/g | 14.2 | 40.8 | NA | 38.5 | 46.4 | NA | 40.4 | NA | NA |

Literature

[1] B. Droz, G. Drouin, L. Maurer, C. Villette, S. Payraudeau, G. Imfeld, Phase Transfer and Biodegradation of Pesticides in Water–Sediment Systems Explored by Compound-Specific Isotope Analysis and Conceptual Modeling, Environ. Sci. Technol. 55 (2021) 4720–4728.

[2] A.J. Williams, C.M. Grulke, J. Edwards, A.D. McEachran, K. Mansouri, N.C. Baker, G. Patlewicz, I. Shah, J.F. Wambaugh, R.S. Judson, The CompTox Chemistry Dashboard: a community data resource for environmental chemistry, J. Cheminform. 9 (2017) 1–27.

[3] M. Arias, M. Paradelo, E. López, J. Simal-Gándara, Influence of pH and soil copper on adsorption of metalaxyl and penconazole by the surface layer of vineyard soils, J. Agric. Food Chem. 54 (2006) 8155–8162.
